# What Makes Happy Counselors? From Self-Esteem and Leader-Member Exchange to Well-Being at Work: The Mediating Role of Need Satisfaction

**DOI:** 10.5964/ejop.v15i4.1881

**Published:** 2019-12-19

**Authors:** Par Eric Dose, Pascale Desrumaux, Jean-Luc Bernaud, Catherine Hellemans

**Affiliations:** a Department of Psychology, University of Lille, Lille, France; b Centre de Recherche sur le Travail et le Développement – CRTTD EA 4132 – INETOP-CNAM, Paris, France; c Laboratoire de Psychologie du Travail et Economique, Université Libre de Bruxelles, Bruxelles, Belgium; University of Bari Aldo Moro, Bari, Italy

**Keywords:** well-being, self-esteem, LMX, need satisfaction, counselors

## Abstract

This study was aimed at examining the extent to which well-being at work is linked to self-esteem and psychosocial resources such as leader-member exchange (LMX). Drawing on self-determination theory, we looked at whether psychological needs (perceived autonomy, competence, and relatedness) act as specific mediators between self-esteem and well-being, and between LMX and well-being. Two hundred and twenty four employment counselors (psychologists) from a French national employment office (Pôle emploi) answered a questionnaire. The data were analyzed using Hayes and Preacher’s method for testing multiple mediations. The results showed that satisfaction of psychological needs for autonomy, competence and relatedness mediated the links between self-esteem and LMX as inductors, and well-being as a criterion. These findings confirm the relevance of self-esteem and LMX for counselors, and of the importance of need satisfaction that plays a critical role in matters of well-being.

Taking into account factors that influence the dimensions of well-being, a growing body of empirical research supports the view that psychological need (PN) satisfaction can strongly affect well-being (Deci et al., 2001). According self-determination theory, needs are universal necessities, for they are essential ingredients of optimal human development and integrity (Ryan, Sheldon, Kasser, & Deci, 1996). In line with this reasoning, need satisfaction promotes psychological health and positive states. The literature has shown that the satisfaction of three needs (competence, relatedness, and autonomy) is positively correlated with various indicators of psychological well-being (Baard, Deci, & Ryan, 2004; Gagné & Deci, 2005; Van den Broeck, Vansteenkiste, De Witte, & Lens, 2008). Pioneering studies by Lévesque, Blais, and Hess (2004) found evidence of a link between well-being and satisfaction of basic needs (Deci & Ryan, 2000, 2008). When looking at factors that may influence dimensions of psychological well-being, the literature on well-being recommend including PN satisfaction. By examining need satisfaction as a possible mediator between self-esteem and leader-member exchange (LMX), the predictors, and well-being, the criterion measure, our contributions to the literature are threefold. First, the literature is rather scarce concerning counselors’ health problems (Bernaud, Cohen-Scali, & Guichard, 2007), and we do not know if their job can satisfy their psychological needs. Second, with few exceptions, the mediating effects of psychological needs and their links to self-esteem and well-being have not been studied much in regard to careers, vocational guidance, and counselors. Third, many studies on psychological needs focus on personal or organizational causes and do not examine, in the same study, the parallel links between personal and organizational variables and each need. It is important to understand whether two factors that reflect positive relations and human growth, self-esteem and leader-member exchanges, are bases for PN satisfaction at work which can be a lever of good psychological health on the job.

Self-esteem and good LMXs are based on relationships, and according to Graen and Uhl-Bien (1995), the quality of LMX is based on signs like satisfaction expressed toward employees, recognition of their potential, and willingness to promote their efficacy. These signs are also indicators of self-esteem, which refers to a person’s appraisal of his or her value (Bardou & Oubrayrie-Roussel, 2014; Baumeister, Tice, & Hutton, 1989; Rosenberg, 1979), which in turn results from a positive attitude toward oneself. Because leadership-member exchanges involve satisfaction via the facilitation of autonomy, affiliation (with colleagues and leader), implemented competence, it enables access to well-being. In the same vein, high self-esteem (Brown & Marshall, 2006), which includes positive overall esteem, positive feelings of self-worth, and positive self-evaluations, is a strong personal component for satisfying the three psychological needs.

Self-esteem can therefore be a basis for personal growth through recognition of oneself as being capable of improvement over time and for seeking fulfillment of one’s potential (competence) through self-confidence in one’s ability to work autonomously and to maintain warm and trusting relationships with colleagues. It is important to add that a second positive pillar, positive psychosocial exchanges at work (LMX), will participate in this mechanism because personal growth cannot evolve without others.

## Well-being and Self-Esteem

Self-esteem is an evaluation of oneself that depends on the person’s identity, and that fluctuates with age and psychosocial transitions. Self-esteem refers to an individual’s view of his or her own value as a person (Coopersmith, 1967), or “the individual’s positive or negative attitude toward the self as a totality” (Rosenberg, Schooler, Schoenbach, & Rosenberg, 1995, p. 141). According to Rosenberg (1979), the self-concept is “the totality of an *individual’s thoughts* and *feelings* having *reference* to *himself* as an *object”* (1979, p. 7). Referring to the self-assessment that an individual makes of his or her own value as a person (Coopersmith, 1967), self-esteem includes a single affective evaluative dimension (Bardou & Oubrayrie-Roussel, 2014; Baumeister et al., 1989; Brown & Marshall, 2006). Although certain scholars consider self-esteem to be multidimensional, most studies a unidimensional approach and an overall measure (Rosenberg, 1985; Vallières & Vallerand, 1990).

Good self-esteem favors social integration, productive behaviors, and high performance at work (Brockner, 1983; Judge & Bono, 2001), a feeling of competence (Bardou & Oubrayrie-Roussel, 2014; Bowling, Eschleman, Wang, Kirkendall, & Alarcon, 2010), and occupational success (Baumeister, Campbell, Krueger, & Vohs, 2003). Self-esteem has effects on psychological and physical health, and on well-being as well (Baumeister et al., 2003; Oyserman, Bybee, Terry, & Hart-Johnson, 2004). One firmly established finding in the literature is the negative correlation between self-esteem and bad health (Rosenberg, 1985). First, low self-esteem has somatic consequences such as eating disorders and addicting conduct and, is a factor of depression and negative emotions (André, 2006; Zeigler-Hill, 2013). Low self-esteem is also a factor of distress and exhaustion. For example, Makikangas and Kinnunen (2003) found that low levels of self-esteem and optimism had a direct negative effect on emotional exhaustion and psychological distress. And a loss of self-respect is correlated with professional burnout (Janssen, Schaufeli, & Houkes, 1999), particularly among the managers (Leroy-Frémont, Desrumaux, & Moundjiegout, 2014). Accordingly, persons with high self-esteem are considered less affected by job stressors and are thus less affected by the consequences of those stressors (Hobfoll & Freddy, 1993). Self-esteem favors individual well-being and better resistance to stress (Oyserman et al., 2004). It facilitates adaptation through self-fulfillment, as well as the social integration (Bardou & Oubrayrie-Roussel, 2014). Self-esteem is correlated with life satisfaction among managers, and explains their well-being (Leroy-Frémont, Desrumaux, Moundjiegout, & Lapointe, 2014).

Finally, high self-esteem seems to be essential to promoting a satisfactory level of well-being (Diener & Diener, 1995; Paradise & Kernis, 2002; Rosenberg, 1985). Longitudinal studies have shown that SE can have marked positive effects on the life outcomes of the individuals (Orth, Robins, & Widaman, 2012; Trzesniewski et al., 2006). Paradise and Kernis (2002), who examined the relationships between self-esteem (level and stability) and psychological well-being measured on Ryff’s (1989) scale, found main effects of high self-esteem on the six well-being subscales. These studies led us to propose the following hypothesis:

*Hypothesis 1:* Self-esteem is positively related to well-being.

## Well-Being and Leader-Member Exchange

Reflecting the direct and interpersonal exchange between the supervisor (the leader) and the subordinate (the member) (Dansereau, Graen, & Haga, 1975; Graen & Uhl-Bien, 1995), LMX is a one of the factors of well-being (Atkinson, Hill, Matthews, & Morgenson, 2016; Borgonovi, Farr-Wharton, & Trinchero, 2014; Epitropaki & Martin, 1999; Sparr & Sonnentag, 2008). LMX is used to indicate the quality of the supervisor–subordinate relationship. Subordinates reporting high levels of LMX, perceive that their supervisors are satisfied with their work, understand their job problems and needs, recognize their potential, and are willing to help them solve work-related problems (Graen & Uhl-Bien, 1995). According to Graen and Uhl-Bien (1995, p. 238), “LMX is both transactional and transformational: it begins as transactional social exchange and evolves into transformational social exchange.” Subordinates perceiving high LMX feel acknowledged, supported, and trusted by their supervisors. The link between LMX as an organizational resource and well-being need to be tested in the specific context of counselors. Indeed, research has found that counselors are subject to psychosocial risks (e.g., burnout: Thompson, Amatea, & Thompson, 2014; workaholism: Machado, Desrumaux, & Dose, 2015). Counselors in particular may have work-related health problems because of work schedules and fragmented activities (Bernaud et al., 2007). Other studies (e.g., Dose, Desrumaux, Sovet, & De Bosscher, 2018 showed that counselors can have a sense of well-being when their work conditions are good (i.e., objective career success, recognition of the hierarchy). Therefore, we hypothesized that:

*Hypothesis 2:* Leader-Member Exchange (LMX) is positively related to well-being.

## Need Satisfaction as a Mediator

According to self-determination theory (Deci & Ryan, 1985, 2000, 2008), human beings have three basic PNs (autonomy, competence, and relatedness), and satisfaction of those needs is essential to their well-being. The need for autonomy involves the freedom to act within one’s environment, to organize one’s own work, and to make decisions and execute behaviors that are in keeping with one’s will (deCharms, 1968; Deci & Ryan, 1985). The need for competence (White, 1959) is satisfied when people feel they are effective and thus have the resources and capabilities required to achieve their goals. The need for relatedness is satisfied when they feel related to others (i.e., they have consideration for others, who show consideration in return). Empirical studies have confirmed that when all three PNs are satisfied, the individual attains a certain degree of vitality (Ryan & Frederick, 1997) and self-concordance (Sheldon & Elliot, 1999). A deficiency in any one of the three can directly diminish well-being. Satisfaction of these needs is thought to be related to general well-being (Deci & Ryan, 2000) and a recent study revealed that need satisfaction in a work setting was positively related to enjoyment of work (Andreassen, Hetland, & Pallesen, 2010).

The relationship between need satisfaction and well-being at work has been established in many studies (Boudrias et al., 2011, 2014; Desrumaux et al., 2015; Gillet, Fouquereau, Forest, Brunault, & Colombat, 2012). The satisfaction of the three needs appears as positively connected to various indicators of psychological well-being (Baard et al., 2004; Boudrias et al., 2011, 2014; Desrumaux et al., 2015; Dose, 2018; Gillet et al., 2012; Van den Broeck et al., 2008).

*Hypothesis 3:* Satisfaction of the needs for relatedness, competence, and autonomy is related to well-being.

A number of studies have also shown that satisfaction of PNs could be considered as a mediator of the relationship between job resources and positive psychological states (Van Den Broeck et al., 2008). The mediating effects of PNs were found with specific populations, including teachers for well-being at work (Boudrias et al., 2011, 2014; Desrumaux et al., 2015). For example, in a study by Desrumaux et al. (2015) with 298 French teachers, satisfaction of the psychological needs for competence and relatedness fully mediated the relationships between climate and well-being, and only satisfaction of competence mediated the relationship between optimism and well-being. It is therefore possible that other personal-factor effects will be mediated by the satisfaction of the three psychological needs. Study of Dose et al. (2018) with counselors showed that the three PN had a mediating effect between the social and psychological career success, and the psychological well-being while only autonomy and relatedness PNs mediatized the relation between financial career success and psychological well-being.

Indeed, self-esteem effects on well-being can be explained 1) by the fact that a high SE generates a good appraisal of one’s work and environment (e.g., by generating protective, positive affects such as the feeling of being challenged and competitive rather than threatened), and 2) by the fact that SE facilitates social relations, affiliation, and reciprocity (Pyszcynski, Greenberg, Solomon, Arndt, & Schimel, 2004). Generally, SE generates a positive state that will facilitate the satisfaction of the three needs. Given that high SE generates good appraisal of one’s work and environment, we presume that high SE will be linked to high PN satisfaction. So we can guess that satisfaction of each of the three psychological needs will play a mediating role between self-esteem and well-being.

According to self-determination theory (Deci & Ryan, 2000), employees strive to actualize their potential and competence (need for competence), develop their relations and build meaningful and satisfying relationships with colleagues (need for affiliation), and grow and progress toward autonomy at the workplace (need for autonomy). Considering the three faces of self-esteem (Brown & Marshall, 2006), which are positive overall esteem, positive feelings of self-worth, and positive self-evaluations, we can assume that positive self-esteem provide the necessary ingredients for satisfying the major psychological needs. Regarding the need for affiliation, it can be argued that, at work, when the values of self-respect, self-esteem and self-development prevail, members will feel confidence in their colleagues and leaders, and will feel encouraged to develop positive and supportive relationships. As a result, employees are able to develop a positive state of health and well-being. Regarding the need for competence, it can be argued that when persons are appreciated and feel they have intrinsic value for their company, they will feel capable and able to engage in various challenging, stimulating, and difficult tasks. Regarding the need for autonomy, it can be assumed that members who have trust in their ability to solve problems and to work without depending on coworkers are more likely to be involved in decision making (Hetland, Hetland, Andreassen, Pallesen, & Notelaers, 2011), take personal initiatives, and express their true selves (Sonnentag, 2003). Accordingly, we set forth the following hypothesis:

*Hypothesis 4:* Satisfaction of the three PNs mediates the relationships between self-esteem and well-being.

According to Graen and Uhl-Bien (1995, p. 238), “LMX is both transactional and transformational: it begins as transactional social exchange and evolves into transformational social exchange.” With respect to PN, subordinates perceiving high LMX feel acknowledged, supported, and trusted by their supervisors, and self-determination theory predicts that their needs for competence, belongingness and autonomy will be fulfilled (Deci & Ryan, 2000). Thus, high levels of LMX may indicate that supervisors are sources of need satisfaction. Theoretically and empirically, links between transformational leadership and need fulfillment (Hetland et al., 2011) are established. Hetland et al.’s (2011) results showed that both transformational leadership and the transactional behavior of management are significantly related to fulfillment of basic needs. Kovjanic, Schuh, Jonas, Van Quaquebeke, and van Dick (2012) showed that need satisfaction (autonomy, competence, and relatedness) mediates the relationship between transformational leadership and job satisfaction. We propose to analyze an extended view of the positive psychological state by using well-being as criterion measure, leading us to predict that employees who feel important and valued by their supervisors will feel supported at the organizational level and will probably feel that their needs at work are satisfied, and finally will feel high well-being.

Resources have “motivational potential” and lead to increased well-being, through the satisfaction of psychological needs (e.g., with social support satisfying the need for affiliation; self-efficacy satisfying the need for competence) (Bakker & Demerouti, 2007; Boudrias et al., 2011, 2014). According to the eudemonic perspective, well-being is not a state but refers to optimal functioning of persons. Therefore, relationships at work are a pathway for developing well-being at work. The links between LMXs and psychological well-being could function through the mechanism of need satisfaction. First, the need for relatedness can be satisfied through positive professional relationships between an employee and a leader. Being supported by the leader, supporting the leader, feeling that daily presence, daily exchanges, and daily information sharing are important, can be strong sources of satisfaction and of good health on the job. Second, the need for autonomy can be satisfied through interdependent positive relationships between an employee and a leader, and autonomy entails wanting to participate in designing one’s work and the team’s work organization. This implies that the leader facilitate participation in professional and social activities and tasks at work. The expected results of these processes are the development of psychological well-being. Third, receiving compliments and accreditation from the leader for one’s competence at work are strong sources of satisfaction that reinforce occupational well-being. Consequently, we can assume that need satisfaction will have indirect effects for all three PNs.

*Hypothesis 5:* Satisfaction of the three PNs plays a mediating role between LMX, and well-being.

**Figure 1:**
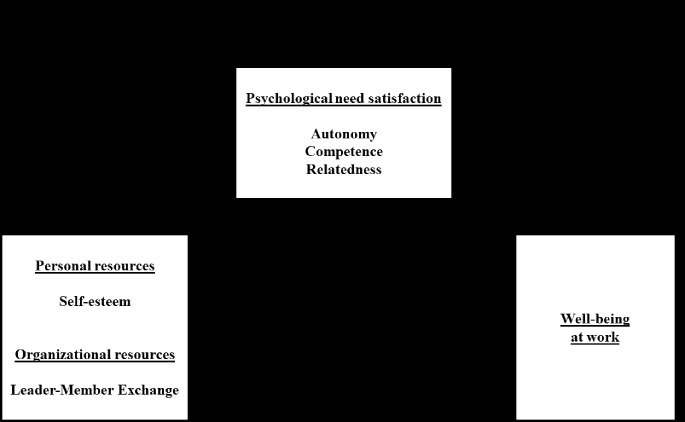
Hypothetical model of psychological well-being at work and psychological need satisfaction among counselors.

In sum, the present study was aimed at verifying our hypotheses using a multivariate approach (Figure 1). We test for the respective effects of each type of predictor (self-esteem as a personal predictor, LMX as a psychosocial predictor) and each mediator (satisfaction of competence, relatedness, and autonomy) on psychological well-being. The relevance of these determinants of well-being was observed on a sample of counselors (psychologists), a professional category known to be subjected to psychosocial risks.

## Method

### Participants

Two hundred and twenty-four professional counselors (97% were psychologists) participated. They all were working in a French national public institution (“Pole-Emploi”) whose role is to follow up onjob seekers and facilitate their employment. They were53men and171women of various ages (*M* = 39.86, *SD* = 10.56), and 4.8 years of work experience ranging. The number of years of seniority at work also varied (*M* = 8.52, *SD* = 8.30). The counselors were working either full time (89.79%) or part time (10.21%). The counselors worked in only one national organization and the sample was homogeneous (French unemployment agency).

### Procedure and Measures

Participants were contacted at work and were asked to fill out an online questionnaire. The questionnaire consisted of 54 items and 20 demographic questions (age, sex, seniority, etc.). The participants filled out the questionnaire at work using seven-point Likert scales ranging from 1 (*totally disagree*) to 7 (*totally agree*). We varied the instructions and response scales in order to minimize response biases (Podsakoff, MacKenzie, Lee, & Podsakoff, 2003). All scales were translated from English into French using a standard translation/back-translation procedure (Brislin, 1980). Cronbach’s α for the study instruments indicated satisfactory internal consistency for all scales.

#### Leader-Member Exchange

Leader-Member Exchange (LMX) was measured using Graen, Novak, and Sommerkamp’s (1982) 7-item LMX 7 instrument. Meta-analytic empirical research has shown that this version of the LMX (e.g., *My manager recognizes my accomplishments and my potential*) provides the soundest psychometric properties and the highest correlations with outcomes, of all available LMX versions (Gerstner & Day, 1997). The seven items (α = .96) were presented as Likert scales with response alternatives ranging from 1 (*strongly disagree*) to 7 (*strongly agree*).

#### Self-Esteem Scale

Rosenberg’s self-esteem scale (Rosenberg, 1979) includes 10 items (α = .87; e.g., *I feel that I have a number of good qualities*) with 7-point Likert-type scales ranging from 1 (*do not agree at all*) to 7 (*completely agree*).

#### Satisfaction of the Three Needs

Satisfaction of the three PNs was measured using Gillet, Rosnet, and Vallerand’s (2008) scale, adapted to work (rather than sports) and validated. Participants were asked to rate 12 items (overall Cronbach’s α = .91) on a 7-point Likert-type scale ranging from 1 (*not at all*) to 7 (*absolutely*) distributed across three categories of four items. Four items measured the need for autonomy (α = .86; e.g., *I often can give my opinion about planning of tasks I have to do*). Four items explored the need for competence (α = .84; e.g., *I often feel very competent*), and four items covered the need for relatedness (α = .81; e.g., *I often feel a lot of sympathy for people with whom I interact*).

#### Well-Being at Work

Dagenais-Desmarais and Savoie’s (2011) measure of psychological well-being at work covered five sub-dimensions (interpersonal fit at work, thriving at work, feeling of competency at work, desire for involvement at work, and perceived recognition at work), with 25 items (α = .96; e.g., *Recently at my job, a smile comes easily to me*). The participants rated the 25 items using a seven-point Likert scale from 1 (*strongly disagree*) to 7 (*strongly agree*).

## Results

The means, standard deviations and, bivariate correlations are presented in Table 1. The correlation matrix gives us a preliminary view of the links between the independent, dependent, and mediating variables. Out of the 15 correlations tested, all were significant and generally went in the expected direction.

**Table 1:** Means, Standard Deviations, and Correlations between the Study Variables

Variable	*M*	*SD*	1	2	3	4	5	6
1. Self-esteem	3.57	1.92	**.87**					
2. LMX	5.46	1.33	.30^**^	**.96**				
3. Autonomy Need Satisfaction	5.14	1.29	.38**	.67^**^	**.86**			
4. Relatedness Need Satisfaction	3.71	1.71	.37^**^	.39^**^	.49^**^	**.81**		
5. Competence Need Satisfaction	5.86	1.14	.43^**^	.57^**^	.71^**^	.58^**^	**.84**	
6. Well-Being	3.57	1.77	.42^**^	.72^**^	.77^**^	.63^**^	.81^**^	**.96**

*Note. N* = 224; Cronbach’s alphas are in boldface along the diagonal.

**p < .01.

For testing the hypotheses, we used Hayes and Preacher’s (2014) SPSS macro, which verifies the existence of direct and indirect links based on regression and non-parametric bootstrapping. We preferred this method over structural equation modeling because the model we tested had too many parameters for our sample (i.e., the ratio of the number of participants to the number of to-be-estimated parameters was less than 20:1) (Kline, 2011). The advocated approach (Hayes & Preacher, 2014) calculates the mediation effect or indirect effect as the product of link A (specific effect of an IV on a mediating variable (MV)) and link B (specific effect of an MV on the DV). Link C represents the total effect of an IV on the DV when the effects of the other IVs are controlled; it is equal to the sum of the direct and indirect effects (Preacher & Hayes, 2008).

In the present study, indirect effects were tested by bootstrapping, which here generated 10,000 alternative samples. One can conclude that a mediation link exists when the confidence interval obtained by way of bootstrapping does not contain zero. The bootstrapping method is robust against potential biases resulting from non-normal data distributions (Preacher & Hayes, 2008).

**Figure 2:**
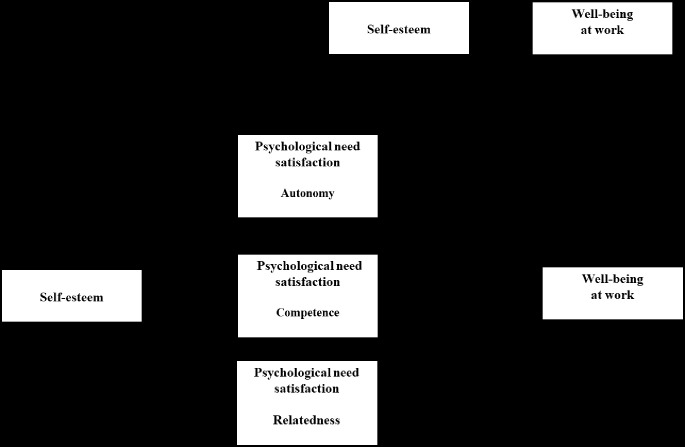
Mediation results with self-esteem (IV) and, well-being as the dependent variable.

**Figure 3:**
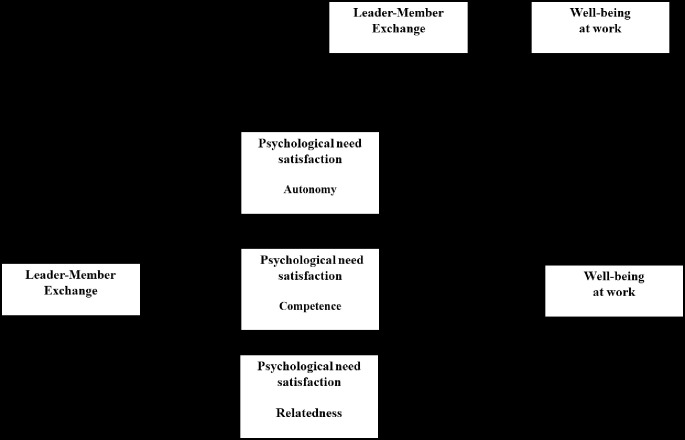
Mediation results with leader-member exchange (IV) and, well-being as the dependent variable.

The effects and links between the variables are presented in Table 2. They show, firstly, that all of the IVs (self-esteem variables and, LMX variables) had significant direct effects on the DV (well-being) (link C’). More specifically, self-esteem and LMX had detrimental effects. All self-esteem and LMX variables significantly promoted need satisfaction (link C’). In Table 2, we can see that link B (satisfaction of the needs for autonomy, competence, and relatedness) had positive effects on well-being. These results showed that satisfaction of the three PNs significantly explains well-being. The results showed that when the indirect effect was factored out of the total effect (link C’), self-esteem and LMX continued to significantly affect well-being. Moreover, the direct effect of these two IVs was weaker than the total effect, suggesting partial mediation. Regarding the indirect effects, the results of the bootstrap analyses (10,000) showed that self-esteem (Figure 2) and LMX (Figure 3) had a significant impact via satisfaction of the three needs.

**Table 2:** Mediation Results with Well-Being as the Dependent Variable

MV	Total Effect (link C)	Effect of IV on MV (link A)	Effect of MV on DV (link B)	Direct Effect (link C’)	Indirect Effect (link C—link C’)	95% CI [LL, UL]
Self-esteem						
Autonomy Need Satisfaction	.43***	.54***	.53***	.14**	.29^a^	[0.05, 0.24]
Competence Need Satisfaction	.43***	.34***	.60***	.23***	.20^a^	[0.13, 0.30]
Relatedness Need Satisfaction	.43***	.46***	.74***	.09*	.35^a^	[0.24, 0.45]
LMX						
Autonomy Need Satisfaction	.47***	.59***	.39***	.24***	.23^a^	[0.18, 0.28]
Competence Need Satisfaction	.47***	.24***	.44***	.37***	.10^a^	[0.06, 0.15]
Relatedness Need Satisfaction	.47***	.40***	.25***	.25***	.22^a^	[0.17, 0.28]

*Note. N* = 224; IV = independent variable; MV = mediating variable; DV = dependent variable; CI = confidence interval; LL = lower limit, UL = upper limit; Bootstrapping *N* = 10,000.

^a^*p* < .05 (bootstrapping 95% CI does not include zero).

*p < .05. **p < .01. ***p < .001.

## Discussion

As hypothesized (H1), our results confirmed that the self-esteem of employment counselors, i.e., their affective or evaluative appraisal of themselves, is linked to their well-being. As many authors have found, self-esteem is positively related to well-being (Baumeister et al., 2003; Leroy-Frémont et al., 2014; Oyserman et al., 2004; Paradise & Kernis, 2002; Rosenberg, 1985). Self-esteem affects well-being in multiple ways. Not only does a high SE generate a good appraisal of one’s own work and of one’s environment, but it also facilitates social relations and reciprocity. In a review of the perspectives on happiness, Ryff (1989) concluded that the recurrent criterion for positive well-being is the individual’s sense of self-acceptance or self-esteem.

A second essential criterion for well-being is exchange. According to Hypothesis 2, our results confirm the relationships between LMX and well-being (Atkinson et al., 2016; Borgonovi et al., 2014; Sparr & Sonnentag, 2008): a high quality of exchange between counselors and their supervisor increase the counselors’ well-being.

The core of our study was to analyze the mediating role of psychological need satisfaction between resources (self-esteem and LMX) and well-being. For that, we began by testing and confirming (H3) that the satisfaction of each psychological need was linked to well-being. As prior studies have shown for persons with different occupations, such as teachers, healthcare professionals, police, sports (Baard et al., 2004; Boudrias et al., 2011, 2014; Deci et al., 2001; Gagné & Deci, 2005), our results revealed that affiliation-need satisfaction was associated with counselors’ well-being at work. Our results also confirmed that competence-need satisfaction is significantly associated with counselor’s well-being at work. Prior research has also shown that employees who believe in their ability to organize and execute their tasks displayed higher levels of well-being (Deci et al., 2001). Lastly, satisfaction of the need for autonomy, which is related to a sense of personal volition and freedom significantly explained well-being at work.

These results, which focus specifically on well-being on the job, are in line with many findings showing that feelings of need satisfaction are related to well-being across many contexts (Boudrias et al., 2011, 2014; Deci & Ryan, 2008; Desrumaux et al., 2015; Gagné & Deci, 2005; Ryan & Deci, 2008, 2017; Sheldon, 2004).

Next, we tested the total, direct, and indirect links between our variables. Our results, which validate Hypothesis 4, yielded indirect effects of relatedness, competence, and autonomy linking self-esteem to well-being. As stated above, self-esteem is a general estimation of the personal value which affects the general vision of the world, the social relations and the work (content, organization, professional relations…). In a study by Graves, Ruderman, Ohlott, and Weber (2012), self-esteem was positively related to enthusiasm, which in turn had a beneficial effect on psychological health. Self-esteem, then, is a necessary ingredient of psychological well-being because it maintains a positive balance at work. Self-esteem is a strength that point out, to the individuals themselves (and to the leader and colleagues), their social value, their ability to implement skills, and their ability to work autonomously and to cope with demands (Leary, Tambor, Terdal, & Downs, 1995). As a result, by satisfying the three needs, self-esteem will enable well-being.

We showed also, confirming H5, that psychological need satisfaction is also a condition that links LMX and well-being. In a more detailed manner, autonomy need satisfaction and relatedness need satisfaction explain a great part (see in direct effect) of the link observed between self-esteem and well-being and also LMX and well-being, and more so than competence need satisfaction. So, it is clearly the satisfaction of social needs that best contributes to understanding the relationship between resources and well-being among counselors. This result is very interesting seeing that the two resources studied are quite different from one another. Indeed, self-esteem is a personal resource, often seen as rather stable, and LMX is a psychosocial resource, in other words, one that depends not only on the individual but also on the psychosocial environment.

To conclude, we learned at least two things from our data. Firstly, it is a good idea to simultaneously analyze the roles of different kinds of resources such as self-esteem and LMX exchanges in order to understand counselors’ well-being. As many psychological health models have shown (e.g., Boudrias et al., 2011), well-being cannot be explained by personal dispositions alone, or by psychosocial factors alone. It necessarily results from two kinds of factors (personal and psychosocial) that optimize our explanation of well-being. In fact, many studies have demonstrated the role of personality in well-being, but our study insists on the fact that a good environment with appropriate leadership is fundamental. Our results showed that having satisfying personal relationships in which empathy and confidence between leader and member are mutually expressed, is a condition for experiencing well-being at work. Secondly, taking need satisfaction into account as a facilitator is a must because doing so enhances the understanding of the well-being drawn from resources. This helps us understand that personal and psychosocial resources have “motivational potential” to increase well-being, through the satisfaction of psychological needs (e.g., social support will help satisfy the “need to belong”). Ensuring the psychological need satisfaction of employees is an important factor that drives well-being at work (Deci, Olafsen, & Ryan, 2017; Gagné & Deci, 2005).

Our findings confirm the eudemonic perspective and the idea that well-being is not a state but refers to optimal psychological experiences and functioning. In this sense, healthy human relationships at work, because they increase self-esteem and positive exchanges, are an avenue for developing well-being. Our study shows that the link between leader member exchange and psychological well-being indeed functions through need satisfaction.

In the introduction, we have justified the relationships between IV and DV for each of the three needs, and followed Ryan and Deci’s point of view (2000, p. 335), which states that: « each need is independently necessary for well-being, and some of our studies, such as that by Reis, Sheldon, Gable, Roscoe, and Ryan (2000) show that each of the three needs does make an independent contribution to the prediction of daily well-being ». All three psychological needs usually gave rise to a statistically significant indirect effect. The mediating effects worked for each of the three needs, but this implies that each one acted as a mediator—although in a singular and complementary manner—and had an indirect effect in the relationships between each IV and DV. It was necessary to test the effects of each need separately because we did not want to combine different complementary needs into an overall score. SDT (Ryan & Deci, 2000) points out the complementarity and singularity of each need, and insists on the fact that if one of the needs is not fulfilled, there will be a negative effect on an individual’s development, well-being, and health at work.

Taken together, the results of this study on counselors provide support for the idea that LMX and self-esteem promote psychological need satisfaction, and that satisfaction of these three needs in turn lead to psychological well-being.

### Limitations

A number of limitations need to be mentioned. Given that this study was based on volunteer participation, one can wonder whether the respondents might not have been those counselors who were the most available and the least stressed, rather than those in difficulty, absent, or ill. Also, self-report questionnaires can favor socially desirable responses. Added to that, all of our information was obtained from the same source. Those two circumstances could create problems related the common variance (Lindell & Whitney, 2001). For future investigations, it might be worthwhile to obtain objective information about the different kinds of social support provided by supervisors, and to take into account of the number of agents assigned to a team, the worker/supervisor ratio, and so on. Lastly, the present cross-sectional study did not allow us to draw any conclusions about causal relations or changes over time in the predictors of well-being. Longitudinal or experimental study designs are needed to test the causal effects of social support and self-esteem on well-being.

### Implications for Practical Applications and Research

Improving the well-being of counselors has many consequences, not only for the counselors themselves but also for jobseekers, applicants, and persons experiencing difficulties at the occupational level. Employment counselors’ well-being is an important contributor to the effective employment of job seekers. Generally, people are motivated to improve or maintain their self-esteem (Pyszczynski, Greenberg, Solomon, Arndt, & Schimel, 2004), because the consequences on their lives are numerous. For counselors, these consequences are extended to people they guide and help. A happy counselor can therefore be more efficient. The conditions for such well-being are related to self-directed attention and especially self-esteem, and to need satisfaction. Superiors also play a major role because supervision help counselors choose their work priorities, decide on their organization, and understand the meaning of their work.

Based on our findings, we can contend that promoting exchanges between supervisors and staff members is critical in increasing well-being. Organizations must seek ways of increasing opportunities to obtain social support for all staff members. Employment agency supervisors play a very important protective role by valorizing, recognizing, and highlighting the capacities and abilities of counselors, and helping them understand their work and tasks by offering them direct assistance with job seekers. Supportive behaviors, such as understanding and acknowledging the employee’s perspective, providing meaningful information, offering opportunities for choice and encouraging initiative taking (e.g., Deci, Connell, & Ryan, 1989) will enhance well-being. If managers are engaged in positive, supporting behaviors (Baard et al., 2004; Deci et al., 2001) and are encouraged and trained to act in a supportive manner (Deci et al., 1989), subordinates will display better physical and psychological well-being. Another question concerns the relationships between well-being and self-esteem. Empirical evidence shows that these variables are very close to each other (Lyubomirsky, Tkach, and DiMatteo, 2006) with various studies revealing moderate to high correlations between measures of happiness and measures of self-esteem. The question of these close links needs to be further explored using different methods (e.g., interviews, experimental methods). In the same way, a closer inspection of the reciprocal influences of LMX and well-being and of well-being and LMX is needed, just as some authors have done for the relationship between job satisfaction and LMX (Volmer, Niessen, Spurk, Linz, & Abele, 2011). Studies that have found that well-being measures affect measures that are similar to LMX (e.g., social support). Future research should explore the temporal dynamics of the LMX–well-being relationship in greater detail.

Further research might also consider potential differences between counselors working in different organizations (guidance of students, guidance of jobseekers, guidance of people seeking career success) with respect to the multiple dimensions of psychological well-being. Among the variables to add to future studies, the organizational climate (Schultz, Ryan, Niemiec, Legate, & Williams, 2014) or the safety climate (Idris, Dollard, Coward, & Dormann, 2012) known to have positive effects on worker psychological health, well-being and performance, could also be tested.
